# A Systems-Based Approach for Cyanide Overproduction by *Bacillus megaterium* for Gold Bioleaching Enhancement

**DOI:** 10.3389/fbioe.2020.00528

**Published:** 2020-06-03

**Authors:** Javad Aminian-Dehkordi, Seyyed Mohammad Mousavi, Sayed-Amir Marashi, Arezou Jafari, Ivan Mijakovic

**Affiliations:** ^1^Biotechnology Group, Department of Chemical Engineering, Tarbiat Modares University, Tehran, Iran; ^2^Department of Biotechnology, College of Science, University of Tehran, Tehran, Iran; ^3^Department of Chemical Engineering, Tarbiat Modares University, Tehran, Iran; ^4^Department of Biology and Biological Engineering, Chalmers University of Technology, Göteborg, Sweden; ^5^Novo Nordisk Foundation Center for Biosustainability, Technical University of Denmark, Lyngby, Denmark

**Keywords:** constraint-based modeling, bioleaching, cyanide, chemically-defined medium, genome-scale metabolic model

## Abstract

With the constant accumulation of electronic waste, extracting precious metals contained therein is becoming a major challenge for sustainable development. *Bacillus megaterium* is currently one of the microbes used for the production of cyanide, which is the main leaching agent for gold recovery. The present study aimed to propose a strategy for metabolic engineering of *B. megaterium* to overproduce cyanide, and thus ameliorate the bioleaching process. For this, we employed constraint-based modeling, running *in silico* simulations on *i*JA1121, the genome-scale metabolic model of *B. megaterium* DSM319. Flux balance analysis (FBA) was initially used to identify amino acids to be added to the culture medium. Considering cyanide as the desired product, we used growth-coupled methods, constrained minimal cut sets (cMCSs) and OptKnock to identify gene inactivation targets. To identify gene overexpression targets, flux scanning based on enforced objective flux (FSEOF) was performed. Further analysis was carried out on the identified targets to determine compounds with beneficial regulatory effects. We have proposed a chemical-defined medium for accelerating cyanide production on the basis of microplate assays to evaluate the components with the greatest improving effects. Accordingly, the cultivation of *B. megaterium* DSM319 in a chemically-defined medium with 5.56 mM glucose as the carbon source, and supplemented with 413 μM cysteine, led to the production of considerably increased amounts of cyanide. Bioleaching experiments were successfully performed in this medium to recover gold and copper from telecommunication printed circuit boards. The results of inductively coupled plasma (ICP) analysis confirmed that gold recovery peaked out at around 55% after 4 days, whereas copper recovery continued to increase for several more days, peaking out at around 85%. To further validate the bioleaching results, FESEM, XRD, FTIR, and EDAX mapping analyses were performed. We concluded that the proposed strategy represents a viable route for improving the performance of the bioleaching processes.

## Introduction

Solid waste is recognized as an important source of metals (Mishra et al., 2008; Rocchetti et al., 2018). Among solid waste, electronic waste (e-waste) is particularly enriched in recoverable precious metals, including gold, silver, and platinum. A number of techniques are currently being used to remove precious metals from e-waste, and among them, bioleaching has shown a lot of potential (Akcil et al., 2015; Zhang and Xu, 2016). Several approaches to improve bioleaching of precious metals have been investigated. Some researchers tested the feasibility of applying sulfate-reducing bacteria to leach gold and copper from e-waste (Kaksonen et al., 2018; Zheng et al., 2018) and a few applied continuous foam fractionation to collect gold or other metals from leaching liquor (Zhou et al., 2020). Although agitation is usually reported as a key factor in bioleaching studies, acceptable metal recoveries were achieved in a bioleaching-without-agitation test, which simplifies the process and offers an economic advantage (Argumedo-Delira et al., 2019). Moreover, two-step processes have recently been proposed to extract metals from e-waste using either cyanide or organic acids and enzymes that react with gold and other precious metals (Işildar et al., 2016; Faramarzi et al., 2020; Marappa et al., 2020).

While cyanide leaching is an efficient method for metal recovery, it is known to be harmful to human health and the environment (Joo et al., 2016). Due to those concerns, bioleaching offers an interesting alternative for the extraction of metals (Pourhossein and Mousavi, 2019), as cyanogenic microorganisms used in bioleaching are able to both produce and consume cyanide (Castric and Strobel, 1969; Knowles and Bunch, 1986). Consumption of cyanide during the process can reduce the risk of human exposure and environmental contamination.

Cyanide can form stable dicyanoaurate complexes with gold ions via an electrochemical process (Kumar et al., 2018). The formation of Au(CN)_2_ complexes occurs as the product of the anodic reaction. This is accompanied by oxygen reduction at the cathode (Kianinia et al., 2018). Equation (1) represents the overall anode-cathode reaction.

$$\begin{array}{l} 4Au+8CN^{-}+O_{2}+2H_{2}O\to 4Au{\left(CN^{-}\right)}_{2}+4OH^{-} \end{array}$$

When cyanide reacts with copper, multiple complexes may be formed. The formation of the Cu(CN)_2_ complex is reported to be the dominant process when cyanide concentration is diminished during the process (Tran et al., 2011). Overall, gold is reported to have less affinity to form complexes with cyanide compared to copper (Breuer et al., 2005; Birich et al., 2019).

Based on the available literature, *Chromobacterium violaceum* (Li et al., 2015), *Bacillus megaterium* (Motaghed et al., 2014), *Pseudomonas aeruginosa* (Biswal et al., 2019), and *Pseudomonas fluorescens* (Li et al., 2015; Potysz et al., 2016) are the most commonly used cyanogenic bacteria for bioleaching of precious metals. Despite multiple studies on the bioleaching of e-waste, the level of cyanide produced by cyanogenic bacteria is still not sufficient to make the process economically viable (Arshadi and Mousavi, 2015). To address this issue, several groups attempted to use metabolic engineering tools to construct strains optimized for cyanide production (Tay et al., 2013). Others focused on the amelioration of bioreactor design (Ghosh and Das, 2018; Marchenko et al., 2018) and optimization of operational parameters, as well as the medium constituents (Saririchi et al., 2012).

In all previous studies on cyanogenic bacteria, to our knowledge, culture media with complex components, e.g., peptone and yeast extract, were utilized. During a fermentation process, every component of the culture medium can potentially affect metabolic fluxes and formation of desired and undesired products (Song et al., 2008). Complex compounds in undefined culture media containing peptone and yeast extract have been reported to result in production of undesired metabolites and decreasing yields of the target metabolites (Zhang et al., 2011). Unlike the undefined medium, a chemically defined medium (CDM) consists only of required nutrients, such as the carbon source, amino acids, lipids, cofactors, metal ions, and vitamins (Hageman et al., 1984). Changes in the composition of a CDM can lead to induction or inactivation of certain cellular functions. Nevertheless, formulation of an optimal CDM for the production of a specified metabolite is a challenging task, when using conventional techniques.

Systems biology tools that are focused on “omics” data and computational methods, consistently surpass conventional approaches when it comes to metabolic engineering and medium design (Biz et al., 2019; Dangi et al., 2019; Cortés et al., 2020). The comparative advantage of systems biology is related to its accounting for the complex relationship between many components of biological systems (Covert et al., 2004; Basu et al., 2018). The basic tools of metabolic systems biology are the genome-scale metabolic models (GEMs), which enable *in silico* studies of metabolic networks. GEMs can generate useful predictions of cellular behavior. These predictions can be used to direct metabolic engineering strategies and formulation of chemically defined media (Covert et al., 2001; Endy, 2005; Terzer et al., 2009; Wang et al., 2017). Several intervention strategies for optimizing fermentation processes have been proposed, e.g., coupling the production of a particular target metabolite with microbial growth (Pharkya and Maranas, 2006; Xu et al., 2011; Klamt and Mahadevan, 2015). GEMs have proved to be equally useful in formulating optimum media for cell culture (Song et al., 2008; Fouladiha et al., 2020).

**Figure 1:**
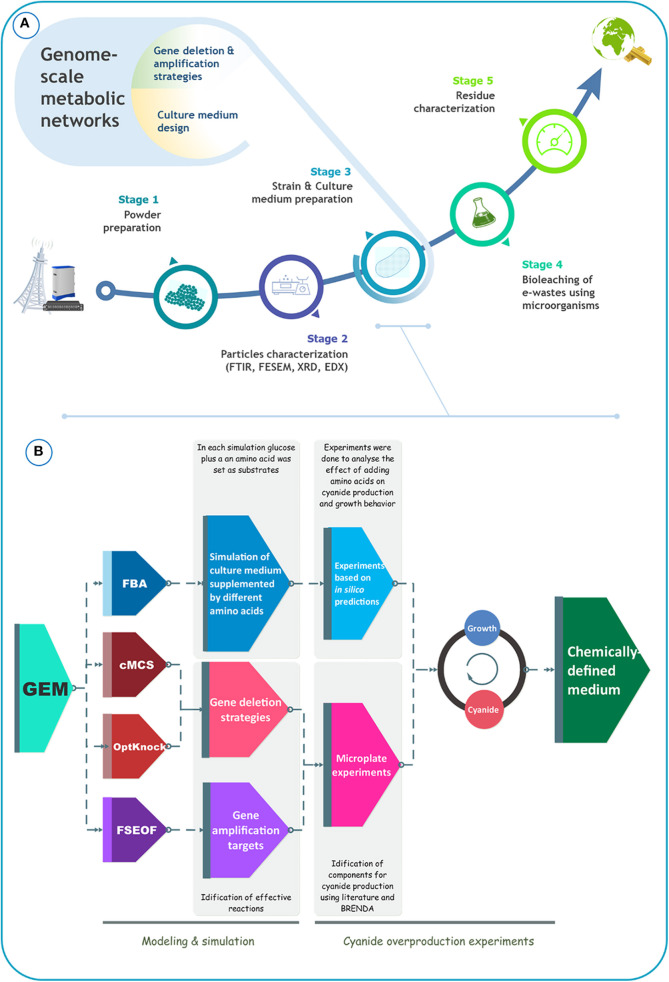
**(A)** Schematical representation of the bioleaching process, **(B)** schematical representation of the procedure that was used to improve cyanide production by *B. megaterium* DSM319.

In the present study, we aimed to boost the production of cyanide by *B. megaterium* DSM319 to improve its suitability for bioleaching of gold from e-waste. For benchmarking, we focused on a specific type of e-waste: telecommunication printed circuit boards (TPCB). Figure 1A schematically depicts the overall process of precious metal recovery from e-waste, with the crucial input of metabolic modeling to enhance bioleaching performance. Our literature survey indicated that the design of the culture medium has been largely ignored in bioleaching studies. Most studies reported the use of common undefined culture media, sometimes improved by adding different amino acids. Herein, we attempted to optimize *B. megaterium* DSM319 growth and also cyanide formation in a defined medium, using an *in silico* GEM-based approach (Figure 1B). Our *in silico* simulations were based on *i*JA1121, the most recent and the most comprehensive GEM for *B. megaterium* DSM319. To identify amino acids to supplement to the culture medium, flux balance analysis (FBA) was used. FSEOF, OptKnock, and constrained minimal cut set (cMCS) analyses were carried out to identify potential targets for gene overexpression or inactivation. Based on these *in silico* simulations, experiments were conceived, that allowed for the formulation of a particular CDM that optimizes cyanide production by *B. megaterium* DSM319. We experimentally tested our strategy for the capacity of the formulated CDM to increase cyanide yield, and consequently improve the effectiveness of gold bioleaching from e-waste. The proposed systems-oriented approach to CDM optimization can be broadly applied to strengthen bioleaching and recovery of metal by other cyanogenic microorganisms.

## Materials and Methods

### Strain and Culture Media

As the seed culture, Nutrient Broth medium, which contains 1 g/L yeast extract, 2 g/L peptone, and 5 g/L NaCl, was inoculated with a single colony of *B. megaterium* DSM319 and cultured overnight in a shaker incubator at 30°C to optical density OD_600_ = 0.7. The mixture was then kept in 2 ml sterile tubes with 0.25 (volume fraction) glycerol stocks at −70°C. To culture *B. megaterium* DSM319, a single tube was added to the 50 ml CDM and maintained in a shaker incubator at 30°C, for approximately 12 h. All materials were either autoclaved at 121°C, or filter sterilized.

For initial experiments, *B. megaterium* DSM319 was cultured in a CDM (hereafter called CDM1) containing 5.56 mM glucose, 37.4 mM NH_4_Cl, 25.9 mM KH_2_PO_4_, 15.1 mM K_2_HPO_4_, 7.26 mM Na_2_HPO_4_.2H_2_O, 5 mM g NaCl, and 0.3 mM K_2_SO_4_. The final experiments were performed using CDM2 containing CDM1, 0.43 mM MgCl_2_.6H_2_O, 0.1 mM MnCl_2_.4H_2_O, 2.5 μm FeSO_4_.7H_2_O, 1.07 μm CoCl_2_, and (82.5–495) μm cysteine. CDM2 with the optimal amount of cysteine (specified in the legend of **Figure 11**) was used for the bioleaching experiments. The experiments were performed at 30°C in 250-ml flasks for 6 h.

### Microplate Assays

Based on *in silico* simulations for identifying gene overexpression and inactivation targets, 32 different components were picked out as potential inhibitors/activators of cyanide production. *B. megaterium* DSM319 was initially grown in the Nutrient Broth for 12 h at 30°C. The *B. megaterium* DSM319 culture was then re-inoculated in CDM1, at OD_600_ around 0.05. Thereafter, 200 μl of the *B. megaterium* DSM319 culture in CDM1 was transferred to each well on a 24-well microplate. Specific metabolic inhibitor/activators were added to each well. The components and their concentrations are listed in Supplementary Table 1. Based on the results obtained through *in silico* simulations (OptKnock, cMCS, and FSEOF) to identify targets for gene overexpression and inactivation, 32 different components were selected as the potential regulators using literature and BRENDA database information. The concentration of the compounds was set as stated in the references. Multiple concentrations were reported for some compounds, so we applied them in two different concentrations to see if a variation in concentration was required. The microplates were incubated at 30°C for 24 h. OD_600_ was monitored to determine bacterial growth, and cyanide content was measured in each well. The microplate assays were performed in triplicate and averaged values were reported.

### Bioleaching Experiments

Discarded TPCBs were used to study gold and copper recovery. To obtain a homogenous powder mixture, wastes were disassembled and pulverized to fine particles as described in the literature (Heydarian et al., 2018). The initial powder was characterized by chemical digestion (Horeh et al., 2016). Bioleaching experiments were carried out in 250 ml flasks containing 50 ml CDM2. The mixtures were then inoculated with a 2 ml stock of *B. megaterium* DSM319 and incubated at 30°C with shaking at 150 rpm. The duration of the bioleaching experiments was 8 days, at a pulp density of 8 g TCDB powder per liter.

### Analytical Procedures

A UV/VIS spectrophotometer (OPTIZEN 320uv) was used to measure OD_600_ of *B. megaterium* DSM319 cultures. A colorimetric glucose oxidase kit (Pars Azmun) was used to determine glucose concentration in the medium. pH and Eh changes were detected by a pH meter (p25, ISTEK) and Eh meter (Metrohm), respectively. The amount of cyanide produced by *B. megaterium* DSM319 was measured using a cyanide meter (Milwaukee). Before cyanide measurements, calibration was performed with predefined standards (0.1, 1, and 10 ppm). For samples with cyanide concentration above 25 ppm, dilutions were performed. To determine the fraction of recovered gold and copper, the bioleaching residues were filtered and analyzed by inductively coupled plasma optical emission spectrometry (ICP-OES). We used X-ray diffraction (XRD) (X'Pert MPD, Philips, the Netherlands) to estimate the component phases of the raw powder using a Co Ka beam at 40 kV and 30 mA. The diffraction angle (2 theta) was set to 10°-90°. Field emission scanning electron microscopy (FESEM) (S-4160, HITACHI, Japan) was utilized to investigate the topological features of the samples at an accelerating voltage of 30-kV. The samples were mounted on adhesive carbon tubes and were coated with a thin gold layer to enhance image quality. To identify functional groups and surface chemical structures of the samples before and after the bioleaching process, we employed Fourier transform infrared (FTIR) spectroscopy (Perkins-Elmer, USA) in a spectral range of 400–4,000 cm^−1^. For chemical characterization and elemental analysis of the samples, energy-dispersive X-ray spectroscopy (EDAX) and mapping analyses were performed, using an EDAX system (BRUKER, Germany). We omitted the gold layer coating on samples since gold is the object of our study.

### *In silico* Simulations

#### Flux Balance Analysis

*i*JA1121, the GEM for *B. megaterium* DSM319 was used for *in silico* analysis (Aminian-Dehkordi et al., 2019). This model includes 1,709 reactions, 1,543 metabolites, and 1,121 genes. FBA was used to simulate the metabolic flux distribution (Liu et al., 2010). FBA uses linear programming to maximize an objective function e.g., the biomass producing reaction in the metabolic network, while assuming no metabolite accumulation during cellular growth (Oberhardt et al., 2009). FBA also contains defined boundaries called environmental conditions. In this approach, the biomass producing reaction is defined as an ancillary reaction that involves the aggregation of biomass constituents (Feist and Palsson, 2010).

#### Growth-Coupled Overproduction Analysis

The coupling of bacterial growth with production of desirable metabolites can be simulated *in silico* (Weber et al., 2015). We used OptKnock as a bi-level programming method to suggest gene deletion strategies leading to metabolite overproduction (Burgard et al., 2003). This optimization tool includes two simultaneous strategies: one which calculates flux distribution based on the maximization of the biomass producing reaction (biomass yield) and the other is bound to the reactions of the former optimization problem by which the desired metabolite flux is maximized. For this optimization method, cyanide production flux was set as the optimization target.

In addition to the OptKnock analysis, we applied the pipeline proposed by von Kamp and Klamt (Klamt and Mahadevan, 2015; Von Kamp and Klamt, 2017) to calculate the cMCSs of the *B. megaterium* DSM319 metabolic network. A cMCS is a set of reactions whose deletion enforces the coupling of cellular growth and the production of the desired metabolite. For this purpose, the SBML (Systems Biology Markup Language) model, *i*JA1121, was converted to a CNA (Cell Net Analyzer) mass flow project. The *i*JA1121 model has 195 exchange reactions by which metabolites can be secreted or consumed. Exchange reactions are pseudo-reactions that describe the exchange of metabolites with the environment. In these simulations, as it is not reasonable to have all these metabolites produced, only the exchange of fermentation products that had been reported in the literature was considered. In our case, production of acetate, formate, lactate, malate, and oxaloacetate was allowed. All spontaneous reactions of the metabolic network that could not be disabled because of their nature were not allowed to be in cut sets, in order to reduce the feasible solution space. In this approach, two distinct matrices for defining inequalities of desired metabolites, $r_{p}-Y_{\min}^{P/S}.r_{s}\leq 0$, and undesired metabolites, $Y_{\min}^{P/S}.r_{s}-r_{p}\leq 0$ and $Y_{\min}^{B/S}.r_{s}-\mu \leq 0$, were introduced. Here, *r*_*p*_, $Y_{\min}^{P/S}$, *r*_*s*_, and $Y_{\min}^{B/S}$ refer to the product rate, the minimum demanded product yield, the glucose uptake rate, and the minimum demanded biomass yield. For both matrices, we added limits on glucose uptake rate and ATP maintenance reaction rate as they characterize the context in which the cMCSs are calculated. For these simulations, we set the glucose uptake limit to 10 mmol (g_DW_ h) ^−1^, $Y_{\min}^{B/S}$ to 0.01 g_DW_ (mmol glucose)^−1^, $Y_{\min}^{P/S}$ to 0.3 of the maximum product yield which is obtained by the calculations, and minimum ATP maintenance reaction rate to 3.96 mmol (g_DW_ h) ^−1^. The remaining procedures were carried on based on the pipeline and the final mixed-integer linear programming (MILP) problem was solved using API functions available in CellNetAnalyzer version 2019.1 (von Kamp et al., 2017).

#### Identification of Gene Overexpression Targets

We used the FSEOF algorithm to identify those metabolic network reactions for which a flux increase leads to the overproduction of cyanide in *B. megaterium* DSM319 (Choi et al., 2010). We first simulated the growth behavior of the strain on a glucose minimal medium using FBA with a glucose uptake rate of −10 mmol (g_DW_ h) ^−1^ and set the biomass producing reaction as the objective function. Then, maximum theoretical cyanide production was obtained by setting the cyanide formation reaction in the metabolic network as the objective function. In the next steps, the cyanide formation reaction was raised step-wise to reach 90% of the theoretical maximum. In each step, flux distributions were obtained with the biomass producing reaction as the objective function. For the selection of target reactions, two conditions were taken into account: $\left|v_{j}^{initial}|\langle |v_{j}^{\max}\right|$ and $v_{j}^{\min}\times v_{j}^{\max}\geq 0$ where $v_{j}^{initial}$, $v_{j}^{\max}$ and $v_{j}^{\min}$ are the initial, maximum, and minimum fluxes of reaction *j* during the simulations, respectively.

## Results and Discussion

### The Impact of Amino Acids on Cyanide Production

**Figure 2:**
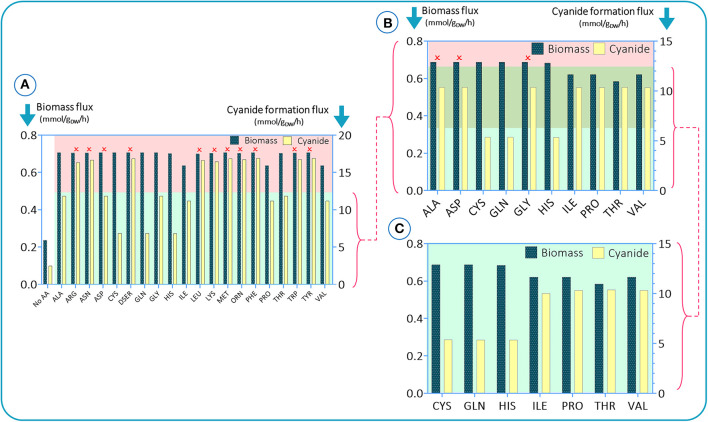
*In silico* results for studying the impacts of amino acid elimination on bacterial growth behavior and cyanide production. In each simulation, a single amino acid is omitted. Simulations were conducted in three steps **(A–C)**. In e. In each step, the amino acids with the highest impacts were selected for the next step.

To investigate the impact of amino acids on growth and cyanide production of *B. megaterium* DSM319, simulations were initially run assuming an amino acid-rich culture medium, i.e., a carbon source and all 20 amino acids. Glucose was defined as the main carbon source by setting the lower limit of its flux to −10 mmol (g_DW_ h) ^−1^ and the uptake fluxes of amino acids were fixed to −5 mmol (g_DW_ h) ^−1^. To assess how each amino acid modulates biomass and cyanide production, the uptake rate of one amino acid at a time was set to zero in each simulation. The results are presented in Figure 2. To sort amino acids based on their essentiality as established by the results of constraint-based modeling, we stipulated a criterion based on the difference between maximum and minimum values of cyanide formation flux in each simulation. The amino acids leading to cyanide production, under the defined criterion (green region), were adopted for the next round of simulations. Initially, there were 10 amino acids with significant impacts on cyanide production. In the next step, the process was repeated to narrow down the list by setting the uptake fluxes of the deleted amino acids to zero [the retained amino acid fluxes were kept to −5 mmol (g_DW_ h) ^−1^]. In Figure 2B, we added another criterion based on the biomass flux maximum and minimum (the top green area). Based on the *in silico* results, alanine, asparagine, and glycine tended to have higher values than the defined criteria and were omitted. Finally, we repeated the last step with the 7 retained amino acids to see if any changes occurred after excluding the other 13 amino acids. Our findings suggest that cysteine, glutamine, and histidine have the most pronounced influence on cyanide formation flux, as their elimination is accompanied by a drop in cyanide production of ~50%. We also observed that isoleucine, proline, threonine, and valine could have an influence on biomass production (Figure 2C).

**Figure 3:**
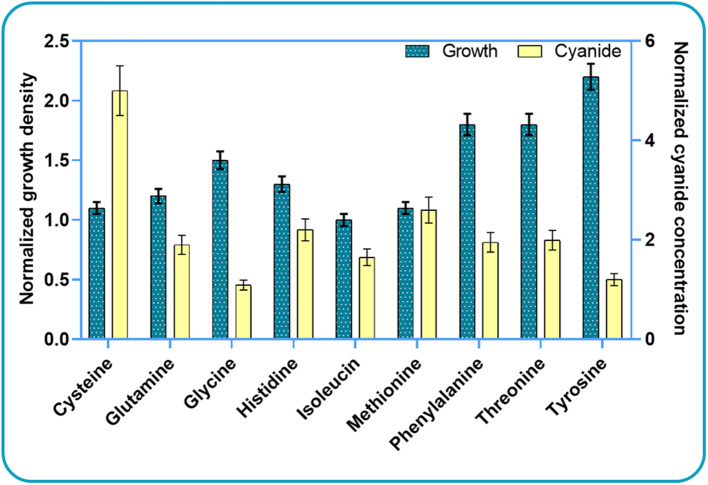
Experimental results for studying the impacts of amino acid elimination on bacterial growth behavior and cyanide production. The growth and cyanide results are normalized with respect to the cell density and cyanide amounts achieved when CDM1 with no amino acid is used as the culture medium.

Based on these results, we further investigated the effect of cysteine, glutamine, histidine, isoleucine, and threonine on bacterial growth and cyanide production. We did not use all high-scoring amino acids for experiments, but rather decided to use some “low-scoring” ones as negative controls. Since phenylalanine and tyrosine did not score “high” *in silico*, we used them as “negative controls” in the experimental analysis. Segregated simulations were performed by utilizing the CDM1 with the addition of a single amino acid in each simulation. In addition to the listed amino acids, we added methionine and glycine to the list. These compounds have been reported as potential components that increase the production of cyanide (Motaghed et al., 2014; Li et al., 2015). Figure 3 illustrates the results of growth behavior and cyanide production after 6 h of segregated simulations. The growth and cyanide results are normalized with respect to the growth and cyanide amount achieved when CDM1 with no amino acid (as the blank experiment) is used as the culture medium, respectively. The addition of each selected amino acid to the CDM1 had a positive impact on bacterial growth. Generally speaking, amino acids could serve as carbon sources which would indeed reinforce the central carbon metabolism and alter the fluxes of alternative pathways, thus triggering formation of subsequent components of biomass. We concluded that tyrosine, threonine, and phenyl-alanine boost bacterial growth the most.

**Figure 4:**
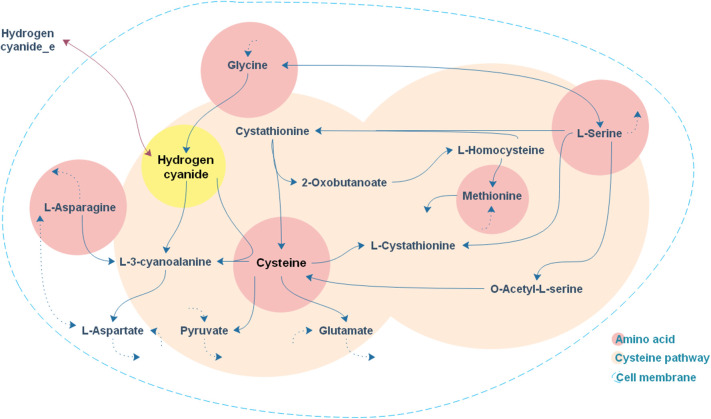
A general overview of the cysteine pathway in accordance with cyanide formation.

As expected, the addition of the selected amino acids to the CDM1 can boost cyanide production. Cyanide production was given a major stimulus, about a 5-fold increase, with the addition of cysteine. The general overview of the cysteine pathway is shown in Figure 4. Additionally, methionine, as reported in some papers (Collins et al., 1980), can be a positive supplement to the CDM1, causing cyanide overproduction. Methionine may influence cyanide formation as a methyl group donor. It may also induce the expression of hydrogen cyanide synthase (Kleid et al., 1992). CDM1 containing histidine, phenylalanine, threonine, and glutamine also increased cyanide amounts, by about 2-fold compared to CDM1 alone. Glycine did not improve cyanide production by *B. megaterium* DSM319, as predicted by the constraint-based model.

### Gene Inactivation Targets

In the next step, gene targets associated with the production of cyanide were identified. Based on the characterization described in Materials and Methods, OptKnock determined target reactions with their associated genes (Supplementary Table 2).

**Figure 5:**
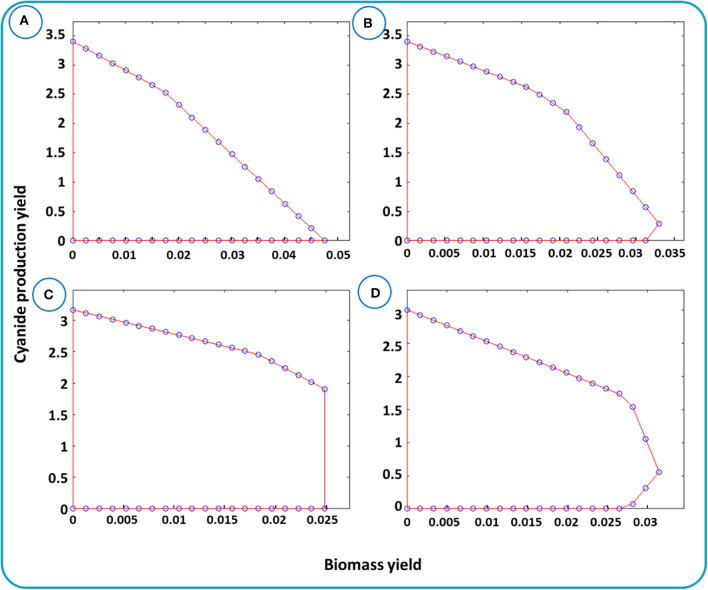
Yield spaces for cyanide production in *B. megaterium* DSM319 evaluated for the metabolic network *i*JA1121. Yield space for cyanide and biomass with the given bounds is presented in **(A)**. Yield space for cyanide and biomass after omitting the reactions raised by cMCSs with μ_*min*_ equals to **(B)** 0.1, **(C)** 0.3, and **(D)** 0.5.

cMCS as an unbiased method is established based on the elementary flux analysis and can be analyzed via yield space analysis. Yield space is a flux polyhedron map that represents the relation between the two yields, e.g., the desired metabolite and biomass. Figure 5 depicts the relationship between biomass and cyanide production yields for different μ_*min*_. μ_*min*_ is the fraction of substrate, here glucose, that is needed to be converted to the desired product, cyanide. In Figure 5A, the maximum cyanide production yield is 3.44 mmol (mmol glucose) ^−1^ and the maximum biomass yield is about 0.05 mmol (mmol glucose) ^−1^. The triangle-like domain shaped in Figure 5A is justified by the principle of conservation of mass. Next, we performed simulations to identify minimal cut sets by setting μ_*min*_ equals to 0.1, 0.3, and 0.5. In each step, the cut sets' reactions were removed from the network and the yield spaces were generated individually. The 3 shapes, Figures 5B–D, describe the trade-off between cyanide and biomass after removing the cut sets. In Figures 5B–D, the maximum biomass yield is smaller than that in Figure 5A, with a reduction of 30–50% due to withdrawing the cut sets from the network. This means that finding strategies to enforce cyanide production while maintaining growth should be possible. Thus, as stated in Materials and Methods, two solutions associated with cMCS with size 6 were applied (Supplementary Table 3).

### Gene Overexpression Targets

**Figure 6:**
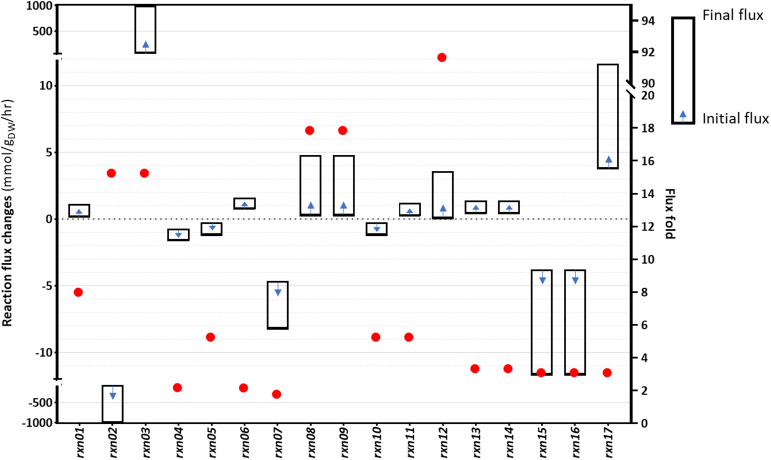
The results of the FSEOF simulation: The identified reactions and their flux changes during simulations.

Although gene overexpression predictions require more arduous operations compared to the identification of gene inactivation targets, sometimes upregulating certain fluxes can improve the production of desired metabolites. We sought to identify fluxes that could be upregulated, with the aim of increasing cyanide production in *B. megaterium*. The problem was defined as an attempt to determine changes of flux distribution while the formation of the desired product is increased, in line with flux scanning based on enforced objective function (FSEOF) (Park et al., 2012). Using the procedure described in Materials and Methods, 17 target reactions were identified as a result of step-wise increments in the cyanide production reaction and the acceptance of up to 9.92% reduction in the biomass producing reaction. In Figure 6, flux changes and fold changes of the gene overexpression targets, as identified by FSEOF, are shown. The details of the reactions and the corresponding information are provided in Supplementary Table 4. In the FSEOF simulation, *rxn12* (conversion of glyceraldehyde to glycerol, involving an NADP^+^ to NADPH regeneration process) had the highest flux amplification, 92-fold. The second-highest flux amplification was obtained for *rxn08* and *rxn09*, about 18-fold, referring to the conversion of fructose1-phosphate to glyceraldehyde and dihydroxyacetone phosphate, and conversion of glucose to fructose, respectively.

**Figure 7:**
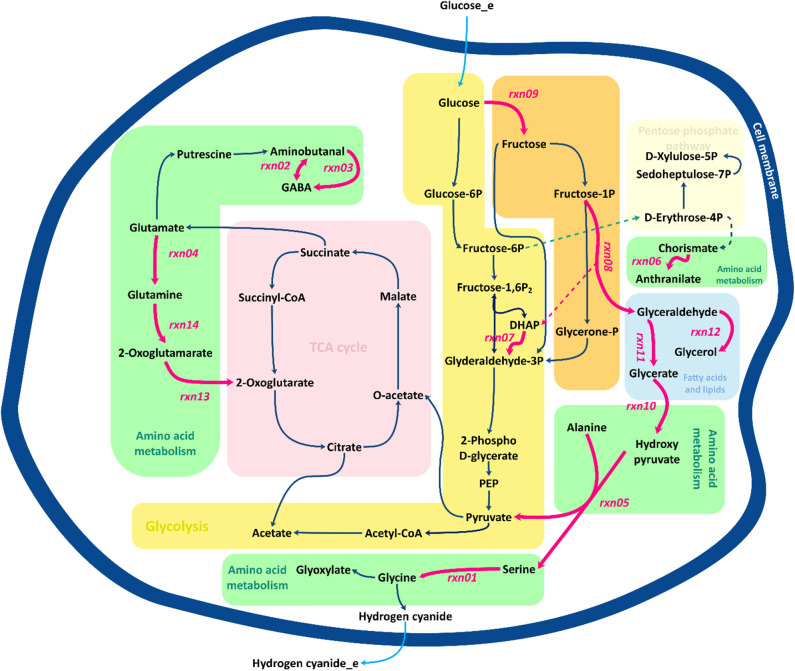
Overview of central metabolic pathways for the amplification of target genes identified by FSEOF. Red arrows identify the reactions that are increased.

Figure 7 presents a schematic description of the identified flux amplification targets. The figure focuses only on the part of the metabolic network that includes the reactions that can be used to define further optimization strategies, as suggested by FSEOF. Glycine is known as the main precursor of cyanide in cyanogenic bacteria, including *B. megaterium*. Biosynthesis of glycine via serine involves several pathways, where glycerate is derived from glyceraldehyde and converted to hydroxy-pyruvate. Fructose 1-phosphate can be converted to glyceraldehyde and dihydroxyacetone phosphate. Here, glyceraldehyde plays an important role as it serves as a source of glycerate and glycerol. Glycerol can either be consumed as one of the sources of dihydroxyacetone phosphate or be converted to glycerol 3-phosphate, which is consumed during the formation of fatty acids. Three identified targets, *rxn03, rxn13*, and *rxn14*, are in the 2-oxoglutarate pathway. Glutamine derived from glutamate can be converted to alanine and 2-oxoglutamarate that is an source of 2-oxoglutarate, a main constituent of the tricarboxylic acid (TCA) cycle. The TCA cycle oxidizes pyruvate to generate chemical energy for the metabolism by producing ATP, NADH and even FADH_2_.

### Optimizing the Composition of the Growth Medium

Gene inactivation and overexpression targets identified by *in silico* simulations were employed to formulate a CDM suitable for cyanide overproduction. Activators and inhibitors of enzymes of the identified reactions were identified from literature and databases (Supplementary Tables 5–7). Hereafter, the components identified as a result of OptKnock and cMCSs will be called inhibitors, and those identified with the aid of FSEOF will be call activators. Cysteine was identified as an activator for *rxn08*, which converts fructose 1-phosphate to dihydroxyacetone phosphate and glyceraldehyde, which is a source for cyanide production.

**Figure 8:**
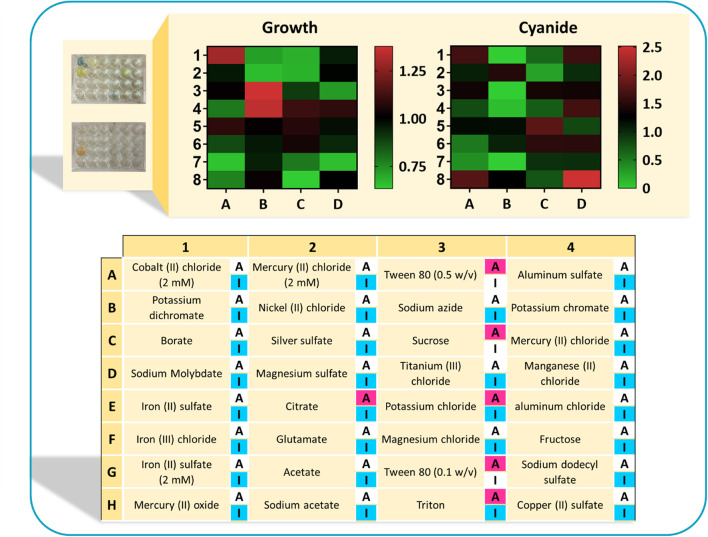
The results of normalized optical density and normalized cyanide concentration in a heat-map view. Each cell represents the measuerments of bacterial growth and cyanide concentration when supplementing the CDM1 medium with a single regulator. Both bacterial growth presented in terms of OD_600_, as well as the cyanide concentrations were normalized with respect to growth and cyanide concentration in the CDM1 medium. Highlighted “A” refers to the potential activators and “I” refers to the potential inhibitors. Some components could have both activatory and inhibitory effects.

In the next step, experiments were conducted to analyze the growth (OD_600_) and cyanide production of *B. megaterium* DSM319 in 6-h experiments, by supplementing available activators and inhibitors separately to the CDM1 medium. The concentrations of regulators were chosen according to values used in published studies (Supplementary Table 1). Figure 8 depicts the results of this assay in a heat-map view. In addition to cysteine with its activatory effect, Tween 80 was identified as a potential activator on the basis of the *in silico* findings. Among the tested compounds, citrate, sucrose and triton have both inhibitory and activatory effects. Other tested compounds were used as potential inhibitors. The findings reveal that 6 compounds are detrimental to *B. megaterium* growth: triton (used as a potential activator), NiCl_2_, NaN_3_, HgCl_2_, sodium dodecyl sulfate, and 5 mM FeSO_4_ (potential inhibitors). Moreover, the components marked as inhibitors may have different impacts on cyanide production, as there are alternative pathways within the cellular metabolism to produce cyanide. By contrast, HgCl_2_, AgSO_4_, MgSO_4_, and acetate restricted cyanide production without inhibiting growth. The best candidates were compounds that enhanced bacterial growth and cyanide production simultaneously. These included MgCl_2_, K_2_SO_4_, FeSO_4_, MnCl_2_, and CoCl_2_. They were added to CDM1 for all subsequent experiments and this optimized culture medium was called CDM2. The microplate experiments showed that CuSO_4_ also enhances cyanide production. Nevertheless, we chose not to add CuSO_4_ to CDM2, as copper is one of the major components in e-waste content and it can influence recovery results.

### The Effect of Cysteine and Other Amino Acids on Cyanide Production

**Figure 9:**
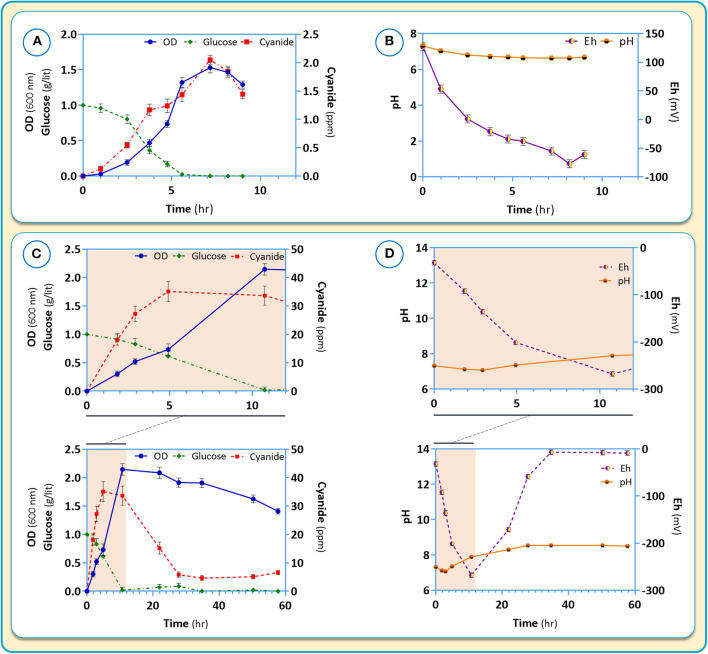
Growth characteristic **(A,C)** and pH as well as Eh **(B,D)** changes for *B. megaterium* DSM319 in different culture media: **(A,B)** CDM2 and **(C,D)** CDM2 + cysteine (2θ μM).

CDM2 was used to further explore the growth and cyanide production of *B. megaterium* DSM319. Independent experiments were carried out using CDM2 supplemented with cysteine (the amino acid with the highest positive impact on cyanide production) (Figure 9). In both C-limited media, no residue of glucose as the main carbon source was observed after 8 h and 10 h in CDM2 and CDM2 supplemented with cysteine, respectively (Figures 9A,C). However, where cysteine as another carbon source was supplemented to CDM2, it took longer for glucose to be consumed. Cellular growth was increased by about 25% in the presence of cysteine. Further, the presence of cysteine in the medium delayed the onset of the stationary phase.

Changes of pH, Eh (oxidation potential) and cyanide production in response to the presence of cysteine in the medium are shown in Figure 9. In the absence of cysteine, the pH is maintained stable around 7 (Figure 9B). Cyanide formation was coupled to the growth of *B. megaterium* DSM319 and peaked at about 2 ppm after 7 h of growth (Figure 9A). During incubation, the negative Eh value decreased to a minimum at 8 hours and began to rise after cyanide reduction.

Upon addition of cysteine to the medium, glucose intake and growth were mildly stimulated. (Figure 9C). The production of cyanide however, rose dramatically, peaking at about 35 ppm after 5 h (Figure 9C). In the presence of cysteine, the medium pH increased to 8.54, which was related to cyanide overproduction. Medium Eh dropped to −268, corresponding to a strong reduction state.

**Figure 10:**
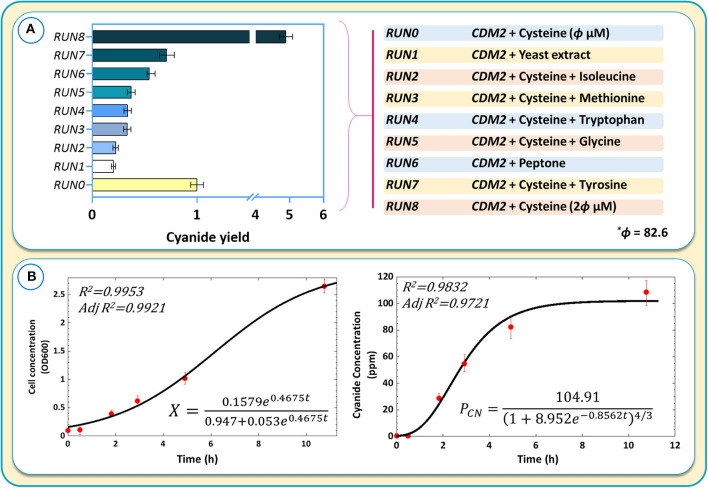
**(A)** The results of cyanide production using different culture media and components of different culture media for cyanide overproduction. Cyanide yields are normalized with respect to the blank sample, *RUN0*. **(B)** Kinetic modeling of cyanide production during the logarithmic growth phase.

Further experiments were performed by adding tryptophan, tyrosine, isoleucine, methionine, and glycine separately to the medium containing CDM2 and cysteine. The purpose was to assess whether any other amino acids, in addition to cysteine, could further boost cyanide production (Figure 10A). The results were normalized with respect to *RUN0* as the blank sample (Figure 10A).

Despite *in silico* simulations that suggested that some amino acids could further increase cyanide production, we found that none of the tested amino acids matched the cyanide productivity achieved with CDM2 and cysteine alone (Figure 10A). Adding complex components, such as yeast extract or peptone, to CDM2 also did not improve cyanide production. The result of *RUN8* confirmed the crucial positive effect of cysteine on cyanide production in *B. megaterium*, where CDM2 with 2θ μM (θ = 82.6) cysteine exhibits optimal cyanide production. Increasing cysteine concentration to 5θ improves cyanide production, while higher concentrations of this amino acid attenuate the production of cyanide. On the other hand, cyanide production is diminished by doubling the amount of glucose at a cysteine concentration of 413 μM.

### Kinetics of Cell Growth and Cyanide Production

To obtain kinetic parameters of cell growth and cyanide production, a revised logistic model (Mu et al., 2006) and an empirical model proposed by Yuan et al. (2018) according to an allometric equation (Enquist et al., 1998) were used. Figure 10B shows the kinetic models obtained for cell growth and cyanide production. As the initial substrate concentration was constant (Mu et al., 2006), the logistic model was able to correctly predict experimental values (*R*^2^ > 0.99). The allometric-based model approximated the cyanide production during the growth phase fairly well, with some more significant deviation from experimental values at higher cyanide concentrations. The presented kinetic models were able to describe the fermentation behavior and the relationship between cell growth and cyanide production.

### Proof of Concept: Bioleaching Experiments

**Figure 11:**
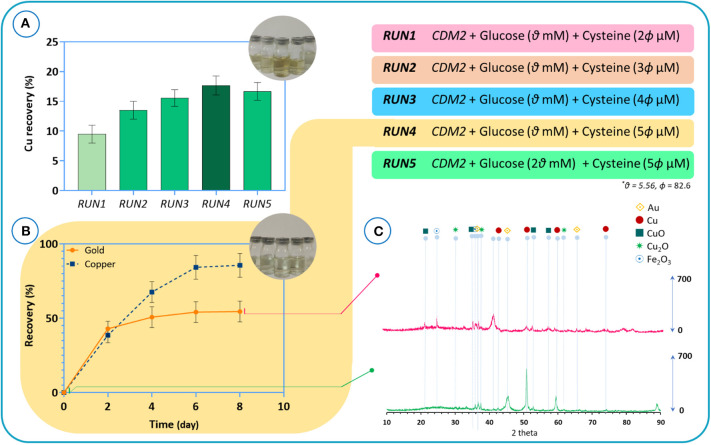
**(A)** The results of copper recovery after 24 h for different culture media, **(B)** the recovery results of gold and copper metals for the bioleaching of TPCB powders by using the *RUN4* medium. Time = 0 refers to the beginning of the bioleaching process, **(C)** XRD patterns of the e-waste before bioleaching and of the residue after bioleaching.

Our findings indicate that, unlike in some other cyanogenic microorganisms where cyanide is produced at the beginning of the stationary phase, cyanide is produced as a primary metabolite in *B. megaterium* DSM319 during the logarithmic growth phase (Niven et al., 1975; Collins et al., 1980; Blumer and Haas, 2000). In the next step, we performed experiments to verify if *B. megaterium* could be used in direct bioleaching systems for treating e-waste. *B. megaterium* DSM319 was cultured in the CDM2 supplemented with cysteine for 6 h. Then, the powder with a pulp density of 8 g/l was added to the bacteria growing in the medium. Experiments were conducted to study the effect of varying cysteine concentrations in CDM2 on the recovery of copper from e-waste powder. The initial powder characterized by chemical digestion illustrated that the raw powder contains 26.7% copper and 665 ppm gold. The results of copper recovery after 24 h are presented in Figure 11A. Copper recovery appears to be directly correlated to cyanide production. Increasing the amount of cysteine boosted cyanide production, which in effect increased copper recovery. To inspect the impact of glucose concentration, we redid *RUN4* by doubling initial glucose concentration – resulting in *RUN5*. As a result, copper recovery declined. This reflects the practical importance of adopting systems biology data in experimental design (Janes et al., 2017). A control assay with CDM2 showed zero recovery of gold and under 10% recovery of copper after 5 days, since the amount of cyanide produced was low. The recoveries of gold and copper in the CDM2 without bacteria were close to zero.

Henceforth, the medium used for *RUN4* was used for all experiments. Figure 11B depicts the results of gold and copper recovery for the bioleaching of TPCB powders. After 4 days, gold recovery peaked out at around 55%, whereas copper recovery kept improving for several more days, peaking out at around 85%. According to the XRD results, before the bioleaching process (Figure 11C), Cu, CuO, and Cu_2_O phase structures are dominant in the TPCB powder. Also, the presence of gold in the sample was demonstrated. The phase analysis of the sample after the bioleaching experiments confirms the ICP results in which the combination of gold and copper structures could hardly be detected. As indicated by FTIR spectra (Supplementary Figure 3), CuO detected at 473 cm^−1^ was mobilized after the bioleaching process. Moreover, the EDAX mapping and composition analysis corroborated these findings (Supplementary Figure 4). The results of EDX analysis consist of spectra with peaks corresponding to the elements that are present in the raw powder before the bioleaching and the residue particles after the bioleaching process. The results indicate that peaks corresponding to gold and copper have diminished after bioleaching. Based on the EDAX mapping, the distribution quality of gold and copper confirmed that the bioleaching process was able to extract gold and copper from the e-waste powder.

### Study of the Kinetic Controls

The bioleaching results emphasize the importance of overproduction of cyanide by cyanogenic bacteria in microbial leaching. They also reflect the valuable contribution of integrated *in silico*-experimental methodology for the design of upscale bioleaching processes. Bioleaching of gold-containing particles has been carried out before, using large stirred bioreactors (Dew et al., 1997). The design of such bioreactors can be facilitated by presenting appropriate logistic or empirical models (Breed and Hansford, 1999).

**Figure 12:**
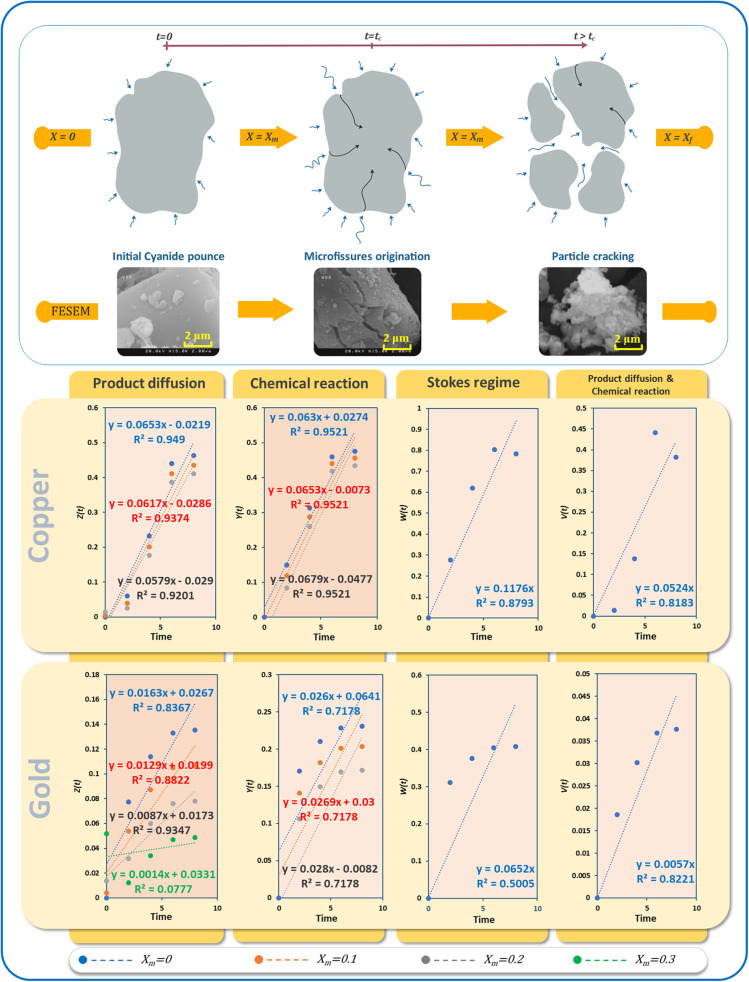
**(A)** Schematic of the crackling core method of e-waste powder bioleaching, different stages visualized by SEM, **(B)** linear regression results for different kinetic models of bioleaching of gold and copper.

Figure 12 shows the progress of the bioleaching process using FESEM imaging (see Supplementary Figure 2 for more details). In the first stage, the powder particles have a smooth surface. In the next stage, micro-fissures and cracks appear, and finally, the particles get broken down into smaller fragments at the end of the bioleaching process. This increases the surface area of particles exposed to the bioleaching agent, in this case, cyanide. The whole process of bioleaching of nonporous particles is typically described by a crackling core model (Martins and Margarido, 1996). In the crackling core model, the particle with size 2R is faced with the fluid containing the bioleaching agent (Figure 12). The interaction of bioleaching agent at the interface leads to the formation of micro-fissures (*t*=*t*_*c*_), which in the presence of high concentrations of the agent leads to particle cracking and sometimes shrinking. The finer particles react with the leaching agent in a fluid environment (Park and Levenspiel, 1975). Under isothermal conditions, the overall leaching rate is equal to the chemical reaction at the interface and its mass transport rate. Letting $\xi =\frac{t_{c}}{t_{total}}$, for swift micro-fissure formation (ξ → 0⇒ *t*_*c*_ → 0), Equations (1, 2) can be applied when kinetics controlled by interfacial chemical reaction and kinetics controlled by diffusion dominate, respectively.

(1) $$\begin{array}{l} t\propto 1-{\left(\frac{1-X}{1-X_{m}}\right)}^{1/3}=Z\left(t\right) \end{array}$$

(2) $$\begin{array}{l} t\propto 1-3{\left(\frac{1-X}{1-X_{m}}\right)}^{2/3}+2\left(\frac{1-X}{1-X_{m}}\right)=Y\left(t\right) \end{array}$$

where *t, X*, and *X*_*m*_ refer to bioleaching time, the fraction of the recovered metal, and the intermediate conversion of metal when particles crack, respectively.

In environments with a high oxygen content, transport of cyanide within the bulk and to the surfaces of particles is limited to either diffusion of cyanide through the product layer or chemical reaction. By contrast, diffusion of cyanide molecules as the bioleaching agent through the bulk is negligible due to the vortexes mixing. Here, we applied kinetic models to examine the role of different rate-controlling steps on the bioleaching process. Letting *X*_*m*_ = 0, 0.1, 0.2 and 0.3, different diagrams were fitted using Equations (1, 2). In addition to film diffusion and the chemical reaction equations based on the crackling core model, we also employed the Stokes regime (Equation 3) (Gomes et al., 2018) which considers liquid boundary layer diffusion, as well as a recently proposed model (Equation 4) based on interface transfer and diffusion across the product layer (Mashifana et al., 2019).

(3) $$\begin{array}{l} 1-{\left(1-X\right)}^{2/3}\propto t=W\left(t\right) \end{array}$$

(4) $$\begin{array}{l} \frac{1}{3}Ln\left(1-X\right)+{\left(1-X\right)}^{2/3}-1\propto t=V\left(t\right) \end{array}$$

Although the product diffusion has a Pearson *R* > 0.95, the results suggest chemical reaction control for the leaching of copper. However, all three fits have identical Pearson correlation coefficients. Thus, we forced the intercepts to zero as another condition. The fitting values indicated that an optimized linear fitting can be obtained using *X*_*m*_ = 0.1 (Supplementary Figure 1). Under these conditions, the rate of copper dissolution is directly related to the availability of unreacted surfaces, as the product layer would offer less resistance.

According to Figure 12, Pearson correlations for gold bioleaching are higher compared to other models when only the diffusion model is applied. Therefore, unlike the previous metal, the bioleaching of TPCB gold metal within the culture medium solution is controlled by product diffusion. The best linear fit for the product diffusion model is obtained for *X*_*m*_ = 0.2. Increasing *X*_*m*_ from zero to 0.2 led to accuracy improvement. However, when increasing *X*_*m*_ to 0.3, a considerable reduction in accuracy was observed.

## Conclusions

Constraint-based modeling tools can be utilized as practical methods to identify genes and associated metabolic reactions that have positive or adverse effects on the production of desired metabolites. Here, we proposed a strategy to overproduce cyanide as the bioleaching agent to improve gold recovery and consequently, bioleaching process efficiency using GEMs. Focusing on the GEM *i*JA1121, FSEOF was used to pick out reactions that redirect the flux toward the production of cyanide. To identify reactions whose inactivation lead to overproduction of cyanide, the OptKnock method was applied. In order to check the feasibility of growth-coupled metabolite production and identify gene targets for the optimization of cyanide production, cMCSs were obtained for the metabolic network. Accordingly, the potential contributing and inhibiting reactions in the metabolic network were identified. Based on these findings, the use of a chemically defined medium containing CDM2 and cysteine was proposed. In this medium, *B. megaterium* DSM319 was able to produce more cyanide than other cyanogenic microorganisms. The results also confirmed that the optimized medium was able to drastically increase cyanide production by *B. megaterium* DSM319. To test the beneficial impact of CDM2 plus cysteine, bioleaching of gold and copper from TPCB was studied. ICP analysis showed that 54% of gold and 78% of copper were recovered. The bioleaching results were further validated by performing FESEM, XRD, EDAX mapping, and FTIR analysis. The crackling pore model and other recent models reported in the literature were considered to identify the main mechanism that controls the bioleaching process. Chemical reaction kinetic control and product diffusion control were found to be dominant for copper and gold extraction, respectively. We would like to emphasize that our strategy uses a comprehensive view of cellular metabolism in order to improve the bioleaching process. The strategy is generic and it can be used to improve several bioleaching processes. This systems-based approach can also be applied to guide genetic engineering of strains (e.g., gene knockouts or overexpression) to make the bioleaching processes more efficient. Development of omics-oriented tools and computational tools has aided researchers in metabolic engineering of microbial strains for a number of biotechnological processes (Campodonico et al., 2016; Nielsen and Keasling, 2016; Suástegui et al., 2017; Martins-Santana et al., 2018; McCloskey et al., 2018). Here we demonstrate that these tools can be equally effective in the bioleaching field.

## Data Availability Statement

All datasets generated for this study are included in the article/Supplementary Material.

## Author Contributions

Conceived and designed the experiments: JA-D, SM, and S-AM. Performed the experiments and analyzed the data: JA-D. Prepared and edited the manuscript: JA-D, SM, IM, S-AM, and AJ. Prepared software and resources: IM and SM. All authors read and approved the manuscript.

## Conflict of Interest

The authors declare that the research was conducted in the absence of any commercial or financial relationships that could be construed as a potential conflict of interest.
