# Supplementary material for: A Systems-Based Approach for Cyanide Overproduction by Bacillus megaterium for Gold Bioleaching Enhancement
Source: Front Bioeng Biotechnol. 2020 Jun 3;8:528. doi: 10.3389/fbioe.2020.00528 (PMC7283520; doi:10.3389/fbioe.2020.00528)
Supplement: Supplementary file 1 [file Presentation_1.PDF]

Supplementary Material

1 Microplate Assays

Based on the results obtained through *in silico* simulations (OptKnock, cMCS, and FSEOF) to identify targets for gene overexpression and inactivation, 32 different components were selected as the potential regulators using literature and BRENDA database information. The concentration of the compounds was set as what was stated in the references. Multiple concentrations were reported for some compounds so we applied them in two different concentrations to see if a variation in concentration is required. The compounds are presented in Table S1.

Supplementary Table S1. List of compounds used for microplate assays.

	**1**	**2**	**3**	**4**
**A**	Cobalt (II) chloride (2 mM)	Mercury (II) chloride (5 mM)	Triton (0.5 w/v)	Aluminum sulfate (1 mM)
**B**	Potassium dichromate (1 mM)	Nickel (II) chloride (1 mM)	Sodium azide (1 mM)	Potassium chromate (1 mM)
**C**	Borate (1 mM)	Silver sulfate (1 mM)	Sucrose (1 mM)	Mercury (II) chloride (1 mM)
**D**	Sodium Molybdate	Magnesium sulfate (1 mM)	Titanium (III) chloride (1 mM)	Manganese (II) chloride (1 mM)
**E**	Iron (II) sulfate (1 mM)	Citrate (1 mM)	Potassium chloride (1 mM)	aluminum chloride (1 mM)
**F**	Iron (III) chloride (1 mM)	Glutamate (1 mM)	Magnesium chloride (1 mM)	Fructose (1 mM)
**G**	Iron (II) sulfate (5 mM)	Acetate (1 mM)	Tween 80 (0.1 w/v)	Sodium dodecyl sulfate (1 mM)
**H**	Mercury (II) oxide (1 mM)	Sodium acetate (1 mM)	Triton	Copper (II) sulfate (1 mM)

2 OptKnock results

Table S2 presents the gene inactivation results obtained by OptKock. Each row represents a single reaction with its relevant genes and EC number which were used as references to determine regulators for the microplate assays. In the table, Subsystem refers to the attributed category that is assigned by the genome-scale model, *i*JA1121.

Supplementary Table S2. List of key reactions and genes identified based on OptKnock. In the reaction column, metabolites' names are abbreviated based on *i*JA1121.

Reaction No.	Reaction	Genes	EC number	Name	Subsystem
1	O2[c] + 2 MBCOA[c] -> 2 H2O[c] + 2 MCRCOA[c]	BMD_2315	1.3.8.7	acyl-CoA dehydrogenase	Amino acid metabolism
2	ATP[c] + F6P[c] -> H[c] + ADP[c] + FDP[c]	BMD_3977	2.7.1.11	6-phosphofructokinase	Carbohydrate metabolism
3	NADP[c] + C01172[c] -> H[c] + NADPH[c] + D6PGL[c]	BMD_5196, BMD_3268, BMD_1913	1.1.1.49, 1.1.1.336	glucose-6-phosphate 1-dehydrogenase	Carbohydrate metabolism
4	NADP[c] + D6PGC[c] -> CO2[c] + NADPH[c] + RL5P[c]	BMD_5197, BMD_3266, BMD_0753	1.1.1.44, 1.1.1.343	6-phosphogluconate dehydrogenase	Carbohydrate metabolism
5	H2O[c] + ACCOA[c] + GLX[c] -> H[c] + COA[c] + MAL[c]	BMD_2890	2.3.3.9	malate synthase	Carbohydrate metabolism
6	ATP[c] + DCMP[c] <=> ADP[c] + DCDP[c]	BMD_4332	2.7.4.25	cytidylate kinase	Nucleotide metabolism
7	H2O[c] + H[c] + DC[c] -> NH3[c] + DU[c]	BMD_4535, BMD_2813	3.5.4.5	cytidine deaminase	Nucleotide metabolism
8	CYTD[c] + DTTP[c] -> H[c] + CMP[c] + DTDP[c]	BMD_4589	2.7.1.48	uridine kinase	Nucleotide metabolism
9	PYR[c] + 3 H[c] + COA[c] + 2 OFER[c] -> CO2[c] + ACCOA[c] + 2 RFER[c]	BMD_4101, BMD_4100	1.2.7.11	2-oxoacid oxidoreductase (ferredoxin)	Carbohydrate metabolism
10	ATP[c] + G[c] -> H[c] + ADP[c] + 2PG[c]	BMD_2747	2.7.1.165	glycerate 2-kinase	Carbohydrate metabolism
11	NAD[c] + SOT[c] <=> NADH[c] + H[c] + FRU[c]	BMD_3588	1.1.1.14	NAD+-dependent sorbitol dehydrogenase	Carbohydrate metabolism

3 cMCS results

Table S3 presents the gene inactivation results obtained by cMCS. Each row represents a single reaction with its relevant genes and EC number which were used as references to determine regulators for the microplate assays. In the table, Subsystem refers to the attributed category that is assigned by the genome-scale model, *i*JA1121.

Supplementary Table S3. List of key reactions and genes identified based on cMCS. In the reaction column, metabolites' names are abbreviated based on *i*JA1121.

Reaction No.	Reaction	Genes	EC number	Name	Subsystem
1	ASER[c] + H2S[c] -> H[c] + AC[c] + CYS[c]	BMD_4826, BMD_0092	2.5.1.47	cysteine synthase A (cysK)	Amino acid metabolism
2	H2O[c] + OA[c] + ACCOA[c] <=> H[c] + COA[c] + CIT[c]	BMD_4756 , BMD_3077	2.3.3.1	citrate synthase (gltA)	Carbohydrate metabolism
3	ATP[c] + SUCC[c] + COA[c] <=> ADP[c] + PI[c] + SUCCOA[c]	BMD_4192, BMD_4191	6.2.1.5	succinyl-CoA synthetase	Carbohydrate metabolism
4	ATP[c] + BUAC[c] -> ADP[c] + BUTP[c]	BMD_4427	2.7.2.7	butyrate kinase	Carbohydrate metabolism
5	FAD[c] + GL3P[c] -> FADH2[c] + T3P2[c]	BMD_0535	1.1.5.3	glycerol-3-phosphate dehydrogenase	Energy metabolism
6	FAD[c] + MAL[c] -> OA[c] + FADH2[c]	BMD_2731	1.1.5.4	malate dehydrogenase	Energy metabolism
7	NADH[c] + H[c] + MQN8[c] -> NAD[c] + MQL8[c]	BMD_0918, BMD_2478	1.6.5.2	NADH dehydrogenase (menaquinone-8 & 0 protons)	Energy metabolism
8	FAD[c] + PMTCOA[c] -> FADH2[c] + C16DCOA[c]	BMD_2315	1.3.8.7	Palmitoyl-CoA:oxygen 2-oxidoreductase	Fatty acid and Lipid
9	COA[c] + C10OCOA[c] <=> ACCOA[c] + C80COA[c]	BMD_4991	2.3.1.16	acetyl-CoA acyltransferase (fadA)	Fatty acid and Lipid

4 FSEOF Results

Table S4 presents the gene amplification results obtained by FSEOF. Each row represents a single reaction with its relevant genes and EC number which were used as references to determine regulators for the microplate assays. In the table, Subsystem refers to the attributed category that is assigned by the genome-scale model, *i*JA1121.

Supplementary Table S4. List of key reactions and genes identified based on the FSEOF simulation. In the reaction column, metabolites' names are abbreviated based on *i*JA1121.

Reaction ID	Reaction	Genes	EC number	Name	Subsystem
rxn01	SER[c] + THF[c] <=> H2O[c] + GLY[c] + METTHF[c]	BMD_5146	2.1.2.1	glycine hydroxymethyl transferase	Amino acid metabolism
rxn02	NAD[c] + H2O[c] + ABAL[c] <=> NADH[c] + 2 H[c] + GABA[c]	BMD_3376 or BMD_2096 or BMD_1546	1.2.1.3	aldehyde dehydrogenase (NAD+)	Amino acid metabolism
rxn03	H2O[c] + NADP[c] + ABAL[c] -> 2 H[c] + GABA[c] + NADPH[c]	BMD_3376 or BMD_2096 or BMD_1546	1.2.1.3	aldehyde dehydrogenase (NAD+)	Amino acid metabolism
rxn04	GLN[c] + CHOR[c] <=> PYR[c] + H[c] + GLU[c] + AN[c]	BMD_4310	4.1.3.27	anthranilate synthase component I	Amino acid metabolism
rxn05	PYR[c] + SER[c] <=> ALA[c] + HPYR[c]	BMD_3035	2.6.1.51	L-Serine: pyruvate aminotransferase	Amino acid metabolism
rxn06	NH3[c] + CHOR[c] <=> H2O[c] + PYR[c] + H[c] + AN[c]	BMD_4310	4.1.3.27	anthranilate synthase component I	Amino acid metabolism
rxn07	T3P1[c] <=> T3P2[c]	BMD_5036	5.3.1.1	triosephosphate isomerase (TIM)	Carbohydrate metabolism
rxn08	F1P[c] <=> T3P2[c] + GLYAL[c]	BMD_5161	4.1.2.13	fructose-bisphosphate aldolase	Carbohydrate metabolism
rxn09	GLC[c] <=> FRU[c]	BMD_1858	5.3.1.5	alpha-D-Glucose aldose-ketose-isomerase	Carbohydrate metabolism
rxn10	NADH[c] + H[c] + HPYR[c] <=> NAD[c] + G[c]	BMD_4963	1.1.1.26	glyoxylate reductase	Carbohydrate metabolism
rxn11	NAD[c] + H2O[c] + GLYAL[c] -> NADH[c] + 2 H[c] + G[c]	BMD_3376 or BMD_2096 or BMD_1546	1.2.1.3	aldehyde dehydrogenase (NAD+)	Fatty acid and Lipid
rxn12	H[c] + NADPH[c] + GLYAL[c] <=> NADP[c] + GL[c]	-	1.1.1.21	alcohol dehydrogenase (NADP+)	Fatty acid and Lipid
rxn13	H2O[c] + C00940[c] -> NH3[c] + AKG[c]	BMD_1722 or BMD_1232	3.5.1.3	omega-amidase	Amino acid metabolism
rxn14	PYR[c] + GLN[c] -> ALA[c] + C00940[c]	-	2.6.1.15	glutamine-pyruvate transaminase	Carbohydrate metabolism
rxn15	PYR[c] + THDP[c] <=> CO2[c] + C05125[c]	BMD_1326 and BMD_1327	1.2.4.1	pyruvate dehydrogenase (acetyl-transferring)	Carbohydrate metabolism
rxn16	C15972[c] + C05125[c] <=> THDP[c] + C16255[c]	BMD_1326 or BMD_1327	1.2.4.1	pyruvate dehydrogenase (acetyl-transferring)	Carbohydrate metabolism
rxn17	ACCOA[c] + C15973[c] <=> COA[c] + C16255[c]	BMD_1874 or BMD_1328	2.3.1.12	dihydro lipoyllysine-residue acetyltransferase	Carbohydrate metabolism

5 Inhibitors and Activators

For every reaction determined by OptKnock, cMCS, and FSEOF reported regulators (compounds with activatory or inhibitory effects) were found. Tables S5, S6, and S7 show the regulators based on the findings achieved using OptKnock, cMCS, and FSEOF approaches, respectively.

Supplementary Table S5. List of inhibitors for the reactions identified by OptKnock.

EC number	Gene(s) in *i*JA1121	Inhibitor
2.7.1.11	BMD_3977	$(NH_4)_2SO_4$ inhibits above 50 mM<br>Citrate<br>Cu^{2+}, Hg^{2+}, K^+<br>KCl inhibits above 50 mM<br>Li_2CO_3 about 75% residual activity at 20 mM, about 40% residual activity at 40 mM, about 10% residual activity at 100 mM, less than 3% residual activity at 200 mM<br>Li_2SO_4 about 55% residual activity at 100 mM, about 30% residual activity at 200 mM<br>$Mg^{2+,}$ $NH^{4+,}$ Ni^{2+}<br>NaCl 1 M, 50% inhibition<br>Zn^{2+} 0.001 mM, 28% inhibition
1.1.1.49	BMD_5196 or BMD_3268 or BMD_1913	Ca^{2+} about 90% residual activity at 2 mM<br>Cd^{2+} 1mM, Co^{2+}, Cu^{2+}<br>D-glucose 6-phosphate about 48% inhibition at 5 mM<br>EDTA<br>Fe^{2+} 5 mM, Fe^{3+}, $KMnO_4$, K^+, Li^+, $MgSO_4$, Mn^{2+} 1 mM 56% inhibition, Pb^{2+}, Zn^{2+}
3.5.4.5	BMD_4535	Borate<br>$CuSO_4$ complete inhibition<br>Fe^{2+}, Fe^{3+}<br>Hg^+, $HgCl_2$<br>SDS, Urea<br>Zn^{2+} above 10 mM
2.7.1.48	BMD_4589	KCl
1.3.8.7	BMD_2315	$CuCl_2$, $AgNO_3$
1.2.7.11	BMD_4101 and BMD_4100	KCl 50 mM
2.7.1.165	BMD_2747	$CuCl_2$ 1 mM, Ca^{2+} 10 mM, $HgCl_2$ 1 mM<br>EDTA, Mn^{2+}, Ni^{2+}
1.1.1.44	BMD_5197 or BMD_3266 or BMD_0753	Cd^{2+}, Co^{2+}, Cu^{2+}, Fe^{2+}, Fe^{3+}, Ni^{2+}, Mg^{2+}, Hg^+
1.1.1.14	BMD_3588	Ag^+ for *B. megaaterium*, $AgNO_3$, $Ba(CH_3COO)_2$ 1 mM<br>$CaCl_2$, $CdCl_2$<br>$CoCl_2$ 1 mM<br>$CuSO_4$ 1mM<br>Cysteine inhibits fructose reduction at pH 9.0, stimulates at pH 7.4<br>Cyanide, D-fructose, D-sorbitol, EDTA<br>Fe^{2+} complete inhibition at 0.91 mM, $FeSO_4$ 1 mM<br>$HgCl_2$, KCl, $MgCl_2$, $MgSO_4$ 1 mM, $NiCl_2$ 1 mM<br>$MnSO_4$, $Pb(CH_3COO)_2$, $ZnCl_2$, $ZnSO_4$, Urea

Supplementary Table S6. List of inhibitors for the reactions identified by cMCS.

EC number	Gene(s) in *i*JA1121	Inhibitor
2.5.1.47	BMD_4826 or BMD_0092	$AgNO_3$ 1 mM, 22% inhibition; 1 mM, complete loss of activity<br>Cd^{2+} 55% inhibition at 1 mM<br>Cl^- ($HgCl_2$)<br>Co^{2+} complete inhibition at 1 mM<br>Copper sulfate 0.78 mM<br>$CuSO_4$ 1 mM, 99% loss of activity<br>EDTA 1 mM, 16% inhibition<br>$FeSO_4$ 1 mM, 96% inhibition<br>Ni^{2+} complete inhibition at 1 mM<br>Sulfide<br>$ZnCl_2$ 1 mM 88% loss of activity
2.3.3.1	BMD_4683	$CuSO_4$, Citrate, D-serine, $HgCl_2$, SDS strong inhibition
6.2.1.5	BMD_4192 and BMD_4191	Acetate<br>Ca^{2+} 10 mM, 75% inhibition<br>Co^{2+} up to 1 mM complete inhibition<br>Cu^{2+} 0.1 mM, up to 10 mM complete inhibition<br>Cystine, EDTA<br>Hg^{2+} up to 1 mM complete inhibition<br>Pb^{2+} up to 10 mM complete inhibition<br>SDS 1.0 mM<br>Sodium arsenite, MnO^{4-}<br>$ZnCl_2$ complete inhibition at 1mM
2.7.2.7	BMD_4427	$HgCl_2$ 10 mM, 47% inhibition
1.1.5.3	BMD_0535	$(NH_4)_2MoO_4$<br>$Al_2(SO_4)_3$<br>$Cu^2{}_+$, Hg_{2+}, methanol, NaCl, Triton X100, Urea<br>Zn^{2+} specifically inhibits ferricyanide reduction assay
1.1.5.4	BMD_2731	$CoCl_2$ 79% inhibition at 1 mM<br>$CuCl_2$ completely inhibits the enzyme at 0.01 mM<br>$CuSO_4$ completely inhibits the enzyme at 0.1 mM<br>$MnCl_2$ 86% inhibition at 1 mM<br>NaN_3 65% inhibition at 1 mM<br>$NiSO_4$ 67% inhibition at 1 mM
1.6.5.2	BMD_0918 or BMD_2478	Acid blue 25<br>Cr^{6+} decrease of enzyme mRNA level together with heme oxygenase-1 and glutathione S-transferase Ya<br>Cu^{2+}, EDTA, $HgCl_2$
1.3.8.7	BMD_2315	Acetylene, ethylene
2.3.1.16	BMD_4991	Acetyl-CoA, CoA<br>Cystamine 10 mM, inactivation with a half-life of 0.6 h<br>Mg_{2+} 25 mM, 20% inhibition, Acetic acid
1.11.1.6	BMD_5226 or BMD_3040	Phenol inhibition of catalase activity<br>Histidine 10 mM histidine reduces activity by 19%<br>Cd^{2+} 0.1 mM, $Co^2{}_+$ partial inhibition at 2 mM, Cr2+, Cu2+
1.2.4.4	BMD_4424 and BMD_4425	Pyruvate

Supplementary Table S7. List of activators for the reactions identified by FSEOF.

**EC number**	**Gene(s) in *i*JA1121**	**Activator**
2.1.2.1	BMD_5146	$CO(NH_2)_2$ Shewanella algae<br>EDTA Shewanella algae<br>L-serine Escherichia coli
1.2.1.3	BMD_3376 or<br>BMD_2096 or<br>BMD_1546	2-mercaptoethanol Escherichia coli<br>Acetaldehyde Sus scrofa<br>Butanal Sus scrofa<br>KCl maximum activity at 0.25 M<br>NaCl maximum activity at 0.25 M<br>NH^{4+} *Pseudomonas aeruginosa* - slight activation<br>Triton X-100
4.1.3.27	BMD_4310	Chanoclavine, Elymoclavine, Iindoleacrylic acid, Prephenic acid Claviceps sp
5.3.1.1	BMD_5036	9-methyl-beta-carbolinium ion Bos taurus<br>dithiothreitol Methanococcus maripaludis<br>phosphite Trypanosoma brucei brucei, Gallus gallus
3.5.1.3	BMD_1722 or<br>BMD_1232	Triton X-100 Bacillus subtilis<br>Sodium lauryl sulfate Bacillus subtilis<br>Sodium deoxycholate Bacillus subtilis<br>Methanol Rattus norvegicus<br>Hydroxylamine Rattus norvegicus
1.2.4.1	BMD_1326 and<br>BMD_1327	GTP Bacillus caldolyticus/ 1-10 mM, slight activation
4.1.2.13	BMD_5161	2-mercaptoethanol Mycobacterium tuberculosis<br>2-oxoglutarate Escherichia coli<br>citrate Escherichia coli<br>Cysteine Haloarcula vallismortis/ highest activity at 1 mM cysteine<br>Cys maximal activity only when both 8 mM Cys and 0.1 mM Fe^{2+} are present. Fe^{3+} or Mn^{2+} can replace Fe^{2+}<br>D-glucose Deinococcus radiodurans<br>Ethanol 10% w/v, increases activity by 24%<br>Diphosphate Haloarcula vallismortis<br>Phosphate Haloarcula vallismortis<br>Triton X-100<br>Tween 20 0.5% w/v<br>Tween 80 0.5% w/v
2.6.1.51	BMD_3035	glucagon Rattus norvegicus, mitochondrial enzyme is markedly induced by
5.3.1.5	BMD_1858	D-glucose Glutamicibacter nicotianae/ if D-xylose is the carbon source D-glucose supplementation stimulates formation of xylose isomerase<br>Sucrose Glutamicibacter nicotianae<br>xylitol Escherichia coli

6 Chemical Reaction Model for Copper Recovery

In this analysis, we forced the intercepts to zero as another condition (Figure S1). The fitting values indicated that an optimized linear fitting can be obtained by $X_m = 0.1$

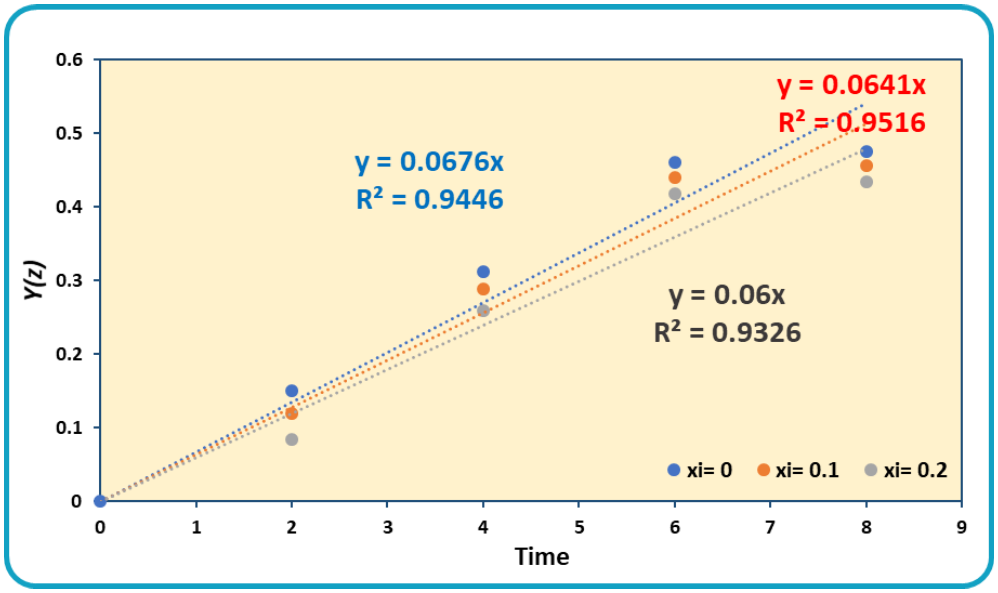

Supplementary Figure S1. Linear regression results using the chemical reaction kinetic model for copper recovery. The intercept is forced to zero for all models.

7 XRD Analysis

We used X-ray diffraction (XRD) (X'Pert MPD, Philips, the Netherlands) to estimate the component phases of the raw powder using a Co Ka beam at 40 kV and 30 mA. The diffraction angle (2 thetas) was set to 10°–90°. In the analysis, the step size was 0.04 with a scanning speed of 0.8 s per step. The degree of crystallinity was measured by Segal's empirical method (Segal et al., 1959). Scherrer's equation was used to determine the crystallite size (Monshi et al., 2012).

8 FESEM Analysis

We used field emission scanning electron microscopy (FESEM) (S-4160, HITACHI, Japan) to investigate the topological features of the samples at a 30-kV accelerating voltage. The samples were mounted on adhesive carbon tubes and were coated with a thin gold layer to enhance image quality.

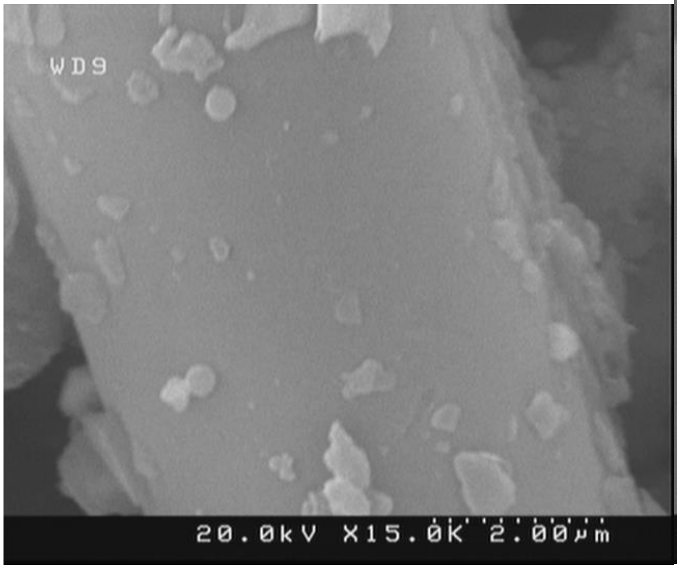

(a)

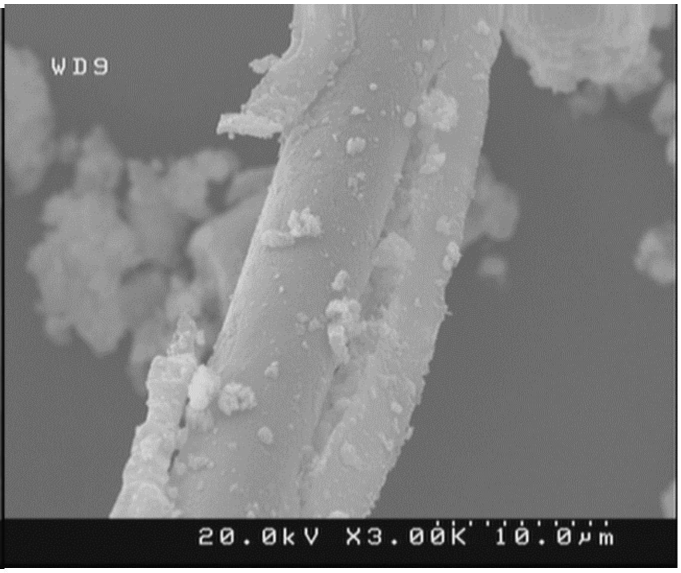

(b)

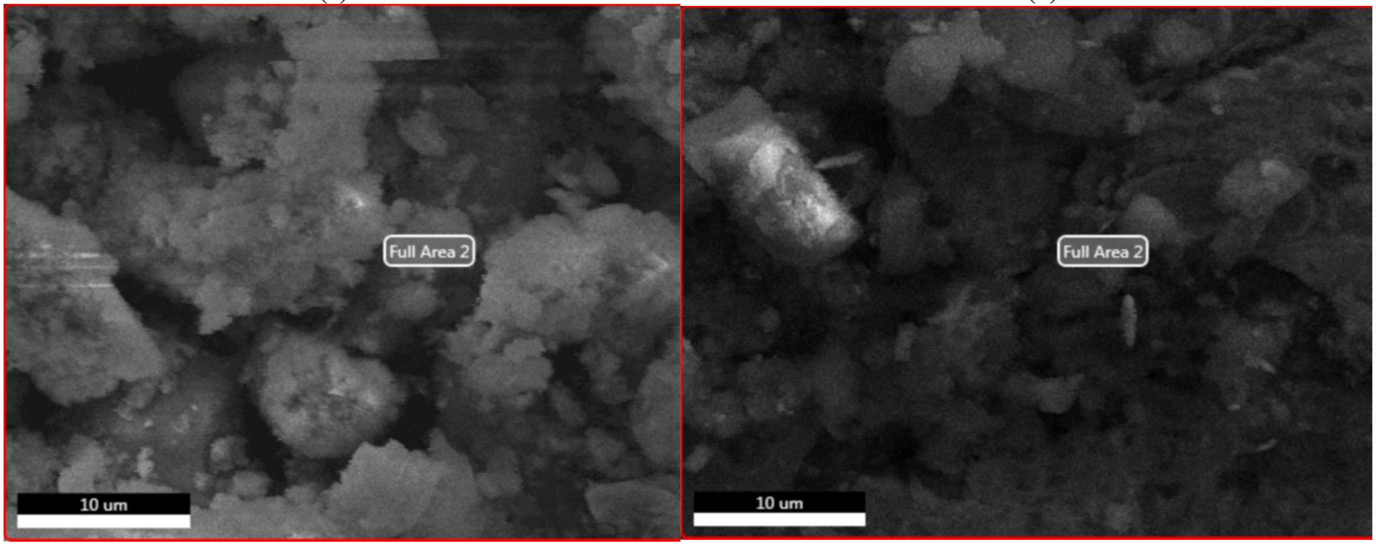

(c)

Supplementary Figure S2- The FESEM images of the powders: surface morphology of particles (a) before bioleaching (with almost smooth surfaces) and (b) after bioleaching (small cracked and broken particles), and (c) distribution size of particles after bioleaching with their irregular morphology

9 FTIR Analysis

To identify functional groups and surface chemical structures of the samples before and after the bioleaching process, we employed Fourier transform infrared (FTIR) spectroscopy (Perkins-Elmer, USA) in a spectral range of 400-4000 per centimeter. Figure S2 depicts the FTIR spectra for the raw sample and the bioleached residue. FTIR spectra provide information about the nature of the structure of the samples. The peak at 473 cm^{-1} was attributed to CuO (Kayani et al., 2015). As can be seen in Figure S2, copper oxide is reduced in amount. The peaks at 1112 cm^{-1} and 1508 cm^{-1} could be associated with C-O, representing alcohol compounds, and N-O, illustrating nitro compounds, respectively. At 1508 cm^{-1}, a C=O stretching from aldehyde/carboxylic acid groups could be observed. 2960 cm^{-1} corresponds to the vibration of C-H groups (alkanes) (Hummel et al., 2005; Geng et al., 2009; Barroso-Bogeat et al., 2014; Yang et al., 2019).

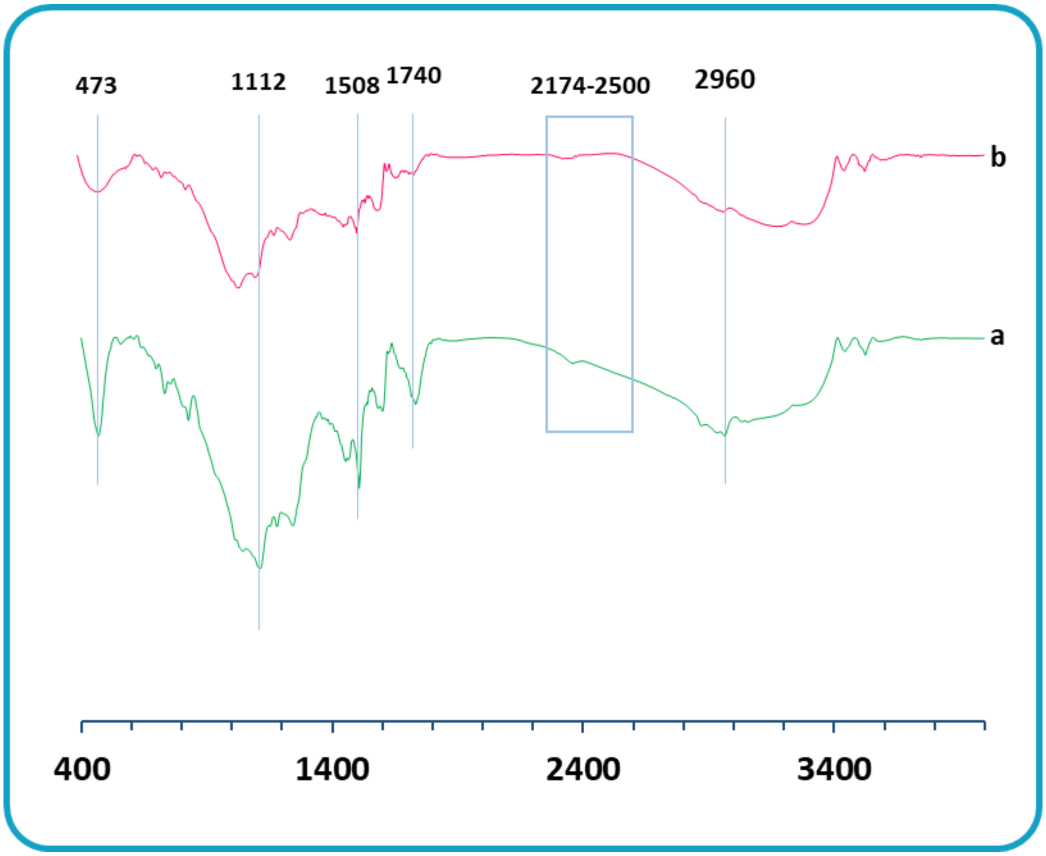

Supplementary Figure S3. FTIR spectra for the sample powder before and after the bioleaching process.

10 EDAX mapping and element analysis

For chemical characterization and elemental analysis of the samples, energy-dispersive X-ray spectroscopy (EDAX) and mapping analyses were used. For this, we used an EDAX system (BRUKER, Germany). We omitted the gold layer coating on samples since gold is the object of our study. Figure S3 represents EDAX mapping and composition characterization of the raw powder and the particles after the bioleaching process.

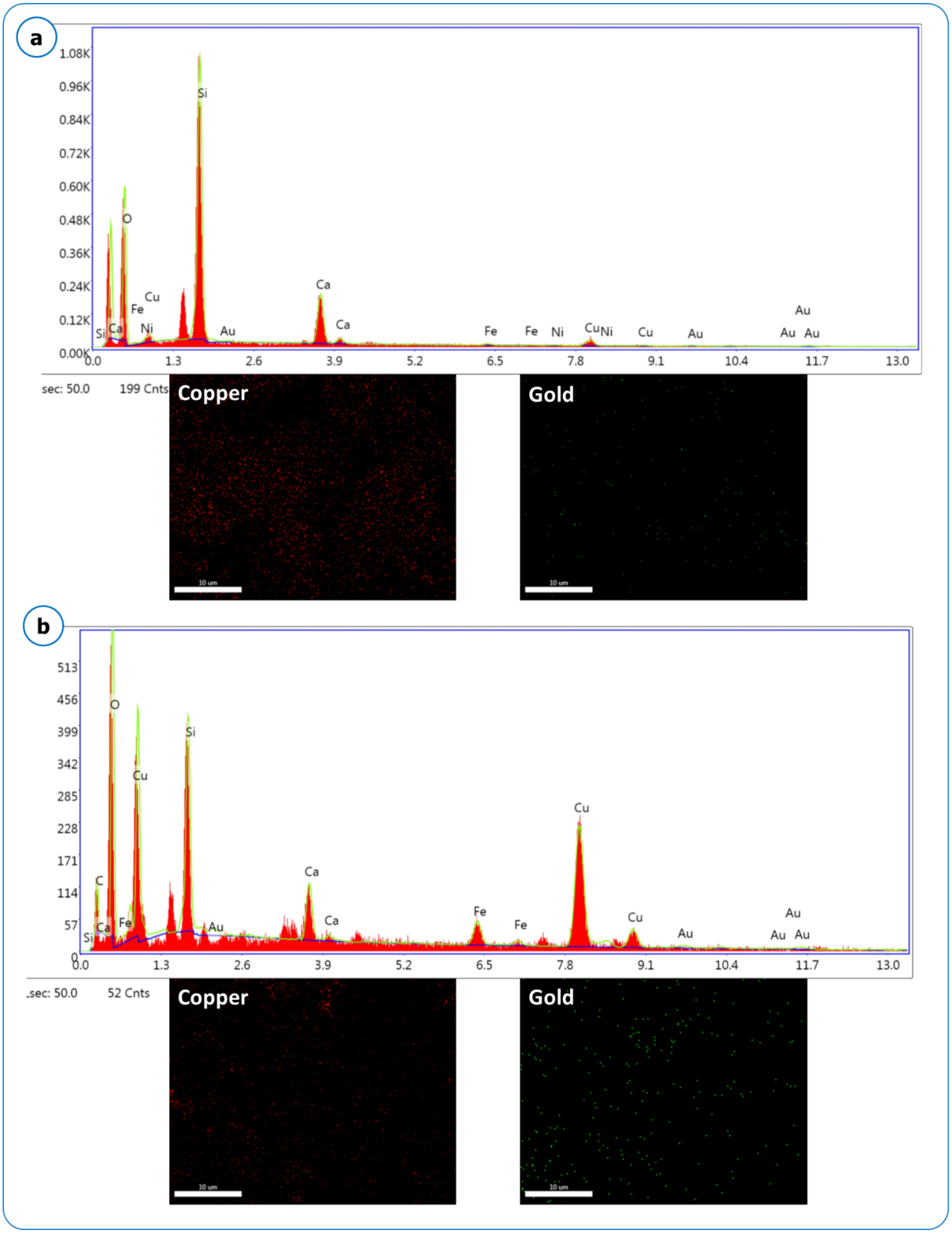

Figure S4. EDAX mapping and composition characterization of (a) pure powder and (b) the particles after the bioleaching process.

References

Barroso-Bogeat, A., Alexandre-Franco, M., Fernández-González, C., and Gómez-Serrano, V. (2014). FT-IR analysis of pyrone and chromene structures in activated carbon. *Energy and Fuels* 28, 4096–4103. doi:10.1021/ef5004733.

Geng, W., Nakajima, T., Takanashi, H., and Ohki, A. (2009). Analysis of carboxyl group in coal and coal aromaticity by Fourier transform infrared (FT-IR) spectrometry. *Fuel* 88, 139–144. doi:10.1016/j.fuel.2008.07.027.

Hummel, P., Oxgaard, J., Goddard, W. A., and Gray, H. B. (2005). Ligand field strengths of carbon monoxide and cyanide in octahedral coordination. *J. Coord. Chem.* 58, 41–45. doi:10.1080/0095897051233132740l.

Kayani, Z. N., Umer, M., Riaz, S., and Naseem, S. (2015). Characterization of Copper Oxide Nanoparticles Fabricated by the Sol–Gel Method. *J. Electron. Mater.* 44, 3704–3709. doi:10.1007/s11664-015-3867-5.

Monshi, A., Foroughi, M. R., and Monshi, M. R. (2012). Modified Scherrer Equation to Estimate More Accurately Nano-Crystallite Size Using XRD. *World J. Nano Sci. Eng.* 02, 154–160. doi:10.4236/wjnse.2012.23020.

Segal, L., Creely, J. J., Martin Jr, A. E., and Conrad, C. M. (1959). An empirical method for estimating the degree of crystallinity of native cellulose using the X-ray diffractometer. *Text. Res. J.* 29, 786–794.

Yang, Y., Lai, M., Zhong, Q., Li, Q., Xu, B., and Jiang, T. (2019). Study on intensification behavior of bismuth ions on gold cyanide leaching. *Metals (Basel).* 9. doi:10.3390/met9030362.
